# On rate-dependent polycrystal deformation: the temperature sensitivity of cold dwell fatigue

**DOI:** 10.1098/rspa.2015.0214

**Published:** 2015-09-08

**Authors:** Zhen Zhang, M. A. Cuddihy, F. P. E. Dunne

**Affiliations:** Department of Materials, Imperial College London, Royal School of Mines, Exhibition Road, London SW7 2AZ, UK

**Keywords:** facet fatigue, microtexture, cold dwell, stress relaxation, hexagonal close-packed, crystal plasticity

## Abstract

A temperature and rate-dependent crystal plasticity framework has been used to examine the temperature sensitivity of stress relaxation, creep and load shedding in model Ti-6Al polycrystal behaviour under dwell fatigue conditions. A temperature close to 120°C is found to lead to the strongest stress redistribution and load shedding, resulting from the coupling between crystallographic slip rate and slip system dislocation hardening. For temperatures in excess of about 230°C, grain-level load shedding from soft to hard grains diminishes because of the more rapid stress relaxation, leading ultimately to the diminution of the load shedding and hence, it is argued, the elimination of the dwell debit. Under conditions of cyclic stress dwell, at temperatures between 20°C and 230°C for which load shedding occurs, the rate-dependent accumulation of local slip by ratcheting is shown to lead to the progressive cycle-by-cycle redistribution of stress from soft to hard grains. This phenomenon is termed *cyclic load shedding* since it also depends on the material's creep response, but develops over and above the well-known dwell load shedding, thus providing an additional rationale for the incubation of facet nucleation.

## 1. Introduction

Dwell sensitivity at 20°C is now well known in titanium alloys and mostly associated with Al-alloyed hexagonal close-packed (HCP) *α*-Ti or near-*α* alloys [1]. The dwell sensitivity of *α*-Ti alloys is believed responsible for some early service failure of discs and fan blades of gas turbine engines. Typical flight operations comprise a single routine loading cycle which includes stress increase during take-off, stress-hold (dwell) when cruising and stress unloading in landing. It is found that load cycles with dwell have a much lower peak stress than load cycles without dwell for the same number of cycles when failure occurs [2]. As a result, the failure in titanium alloys at ambient temperature cannot be simply based on the number of load cycles, and the reduction in cycles to failure because of the stress-hold is known as the dwell debit.

**Figure 1.**
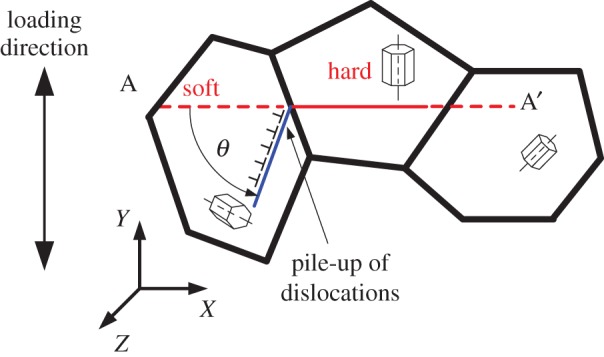
Rogue grain combination.

Much work has been carried out in order to understand the mechanistic basis for the dwell debit [2,3]. This has largely focused on the role of micro-texture, and the combination of very particular crystallographic orientation combinations, operating synergistically with the known creep response of Ti alloys, which occurs even at low temperatures. Work by Sinha *et al.* [4–6] suggested that loading along the {0002} component of the texture enhances dwell fatigue, and that dwell fatigue crack nucleation is associated with the formation of near-{0002} facets that lie close to perpendicular to the loading direction. This observation was supported by the Stroh formulation for crack nucleation [7] in a qualitative model described by Evans & Bache [8,9]. Crystallographic orientation combination of hard and soft grains (that is, HCP grains badly and well orientated for slip, respectively) in a polycrystalline sample was found to be significant in producing large local stresses at grain boundaries [10]. A rogue grain combination, shown schematically in figure 1, with a primary hard grain with *c*-axis orientated about parallel to the local maximum principal stress adjacent to a soft grain was investigated by Dunne *et al.* [10–12], and the resulting grain boundary stresses quantified as a function of the soft grain crystallographic orientation.

Hasija *et al.* [13] were the first to show that ‘cold’ creep occurring in the adjacent soft grain under load hold could lead to the phenomenon of load shedding in which stresses are redistributed into the hard grain during the hold period, potentially dramatically reducing the fatigue life of the material. Dunne *et al.* also reported [11] that loading under a stress controlled hold is more damaging than that under a strain-controlled hold.

Although it is known that cold creep is crucial for facet nucleation, the behaviour has mostly been investigated at a temperature lower than that at which diffusion-mediated deformation is expected to occur. However, some experimental studies have suggested that the ‘cold’ dwell effect diminishes as temperature increases and disappears at around 200°C [14], such that at this temperature and above, a dwell debit is no longer observed. The low temperature (i.e. less than 200°C) creep deformation in alpha and alpha–beta Ti alloys is observed for applied stresses of only about 0.2% yield stress [15]. The mechanistic basis for the diminution of the cold dwell effect on the dwell debit as temperature increases up to about 200°C has remained elusive, but continues to be of crucial importance in aero-engine applications where the same temperature dependence of facet nucleation has also been observed (D Rugg 2014, personal communication). In addition, anecdotally (B Arthurs & A Walker 2014, personal communication), it is known in the aero-engine industry that the dwell debit is worst at some temperature between 90°C and 120°C, but again, mechanistic understanding remains elusive.

Hence, this study aims to address the temperature sensitivity of dwell fatigue, and to present the mechanistic basis for the sensitivity, and for its diminution as the temperature approaches 200°C. As a result of this, it is also possible to identify the worst-case temperature scenario leading to dwell fatigue, which has also been observed through experience within the aero-engine industry (D Rugg 2014, personal communication). The technique employed is that of rate and temperature-dependent crystal plasticity using experimentally informed slip behaviour. The crystal model and its temperature sensitivity are addressed first, with subsequent focus on the temperature dependence of the anisotropic elasticity properties and of the independent HCP Ti basal, prismatic and pyramidal slip system strengths. The crystal model is then employed to examine specifically the local, rogue grain-level stress relaxation and creep response resulting from strain and stress-controlled loading, respectively, over the temperature range of interest, in order to examine systematically their roles in the temperature sensitivity of rogue grain stresses and hence in cold dwell. Finally, the response of oligocrystals under cyclic loading over the range of temperature is examined in order to quantify the stress relaxation and ratcheting behaviours expected to result.

## 2. Crystal plasticity model with temperature-dependent slip rule

The crystal plasticity model used has been well described in earlier papers [10,12,16,17] and is covered only briefly here, but its temperature sensitivity has not previously been explicitly addressed. The key rate-controlling process assumed is that of the pinning and thermally activated release of gliding dislocations giving rise to an overall average dislocation glide velocity which, by virtue of the thermal activation, depends explicitly on temperature and an activation energy, Δ*H*, quantifying the temperature sensitivity [16]. Dislocation pinning is taken to occur through the presence of lattice obstacles, which may include solute atoms, the sessile statistically stored dislocations and their associated structures, as well as geometrically necessary dislocations, incorporated in the slip rule as an overall obstacle density of *ρ*_*o*_. The resultant slip rule [10,12,16,17] determining the slip rate ${\dot{\gamma }}^{\alpha }$ of a given slip system *α* takes the form

2.1 $${\dot{\gamma }}^{\alpha }=\rho_{m}\nu b^{2}\exp\left(-\frac{\Delta H}{k\Theta }\right)\sinh\left[\frac{(\tau^{\alpha }-\tau_{c}^{\alpha })\gamma_{0}b^{2}}{k\Theta \sqrt{\rho_{o}}}\right],$$

where *τ*^*α*^ is the resolved shear stress for the *α*th slip system, *τ*^*α*^_c_ the corresponding critical resolved shear stress (CRSS), *Θ* the temperature, *ρ*_*m*_ the density of mobile dislocations and *γ*_0_ a representative shear strain magnitude. In this equation, *v* is the frequency of attempts of dislocations to jump obstacle energy barriers, *b* Burger's vector magnitude and *k* is the Boltzman constant. The quantity Δ*H*, discussed above, is the activation energy for self-diffusion. The activation energy for self-diffusion and substitutional solute diffusion are remarkably higher than interstitial diffusion, and self-diffusion is known to play a significant role in creep [18]. The activation energy Δ*H* is thought to be temperature independent in low temperature creep [19,20], and the variation of Δ*H* with strain is also argued to be insignificantly small [20,21].

In this study, the temperature dependence of the slip system critical resolved shear stresses and elastic moduli are fully incorporated. Based on Taylor's dislocation model [22], the temperature-dependent critical resolved shear stress is written

2.2 $$\tau_{c}^{\alpha }=\tau_{c0}^{\alpha }(\Theta )+G(\Theta )b\sqrt{\rho_{\mathrm{SSD}}+\rho_{\mathrm{GND}}},$$

in which *ρ*_SSD_ is the density of statistically stored dislocations (SSDs), *ρ*_GND_ the density of geometrically necessary dislocations (GNDs), *b* Burger's vector magnitude and *G* the temperature-dependent shear modulus. $\tau_{c0}^{\alpha }$ is the initial, strain-free, temperature-dependent critical resolved shear stress for given slip system *α*. The SSD density is taken to evolve with the rate of accumulated slip, $\dot{p}$ such that

2.3 $${\dot{\rho }}_{\mathrm{SSD}}=\gamma^{\prime }\dot{p},$$

where *γ*^′^ is the hardening factor, chosen to ensure the experimentally observed hardening is reproduced. The density of GNDs is determined from knowledge of the gradient of the plastic deformation gradient **F**^P^ and has been reported in detail elsewhere (e.g. [10])

2.4 $$\sum_{\alpha =1}^{N_{\;s}}{\text{b}}_{G}^{\alpha }⊗{\rho_{G}}^{\alpha }=\mathrm{curl}({\text{F}}^{P}).$$

The above-equation can be formulated into a set of linear equations of the form

2.5 $$\text{A}\rho_{G}=\Lambda ,$$

where **Λ** contains the components of the gradient of plastic deformation tensor, and **A** that for the net Burger's vector. The GND density at each strain step is given by

2.6 $$\rho_{G}={\left({\text{A}}^{T}\text{A}\right)}^{-1}{\text{A}}^{T}\Lambda$$

which is based on an *L*_2_ minimization in order to solve for a non-unique problem [17,23], resulting from the absence of a unique mapping between lattice curvature and dislocation content.

The slip rule presented in equation (2.1) describes the thermally activated dislocation forward or reverse jumps from two stable positions. This activation event is thermally driven under the applied stress [16]. The sinh term comes from expanding the average glide velocity by considering a symmetrical arrangement in forward and reverse jumps [12]. A feature of the slip rule is that it inherently preserves the thermodynamic argument, by considering *τ*^*α*^−*τ*^*α*^_c_, so that the long range internal stress (or image stress) has a non-zero mean value over the area actually swept out in the slip plane [24]. By considering Taylor hardening as in equation (2.2), work hardening dislocations interact with the elastic fields generated by sessile dislocations and ultimately may themselves become immobilized. Hence the sessile dislocations create back stresses that increase the stress required for deformation. Using an effective plastic strain-dependent *ρ*_SSD_ evolution reasonably reproduces the recovery process, i.e. saturation of strain hardening. The density of statistically stored dislocations remains constant when there is no accumulated plastic strain. Most significantly, the slip rule in (2.1) and (2.2) explicitly relates slip rate and temperature, as well as its dependence on strain hardening.

### (a) Calibration of temperature-dependent properties

The key temperature-dependent properties appearing in the crystal model include the anisotropic elastic constants and the slip system strengths. For the latter, it is well known that for HCP alpha-Ti, the type II *c*+*a* pyramidal systems possess a slip strength significantly higher than that for the a-type systems [25]. Significant experimental property data now exist for the single-crystal and polycrystal response of near-alpha Ti alloy Ti-6Al, and, for this reason, this material is considered in this study. The methodology adopted is to calibrate the model firstly against the room temperature (20°C) single-crystal and polycrystal response, and then to use the results of several experimental studies [13,26,27] in order to incorporate the temperature sensitivity to temperature in excess of 200°C.

**Figure 2.**
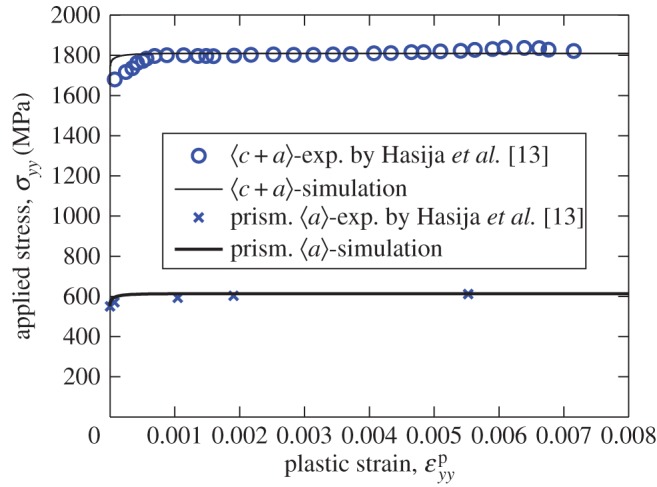
Experimental and crystal model curves for prismatic 〈*a*〉 and pyramidal 〈*c*+*a*〉 slip system response based on single-crystal creep experimental data [13].

Hasija *et al.* [13] obtained stress–strain data for prismatic 〈*a*〉 and pyramidal 〈*c*+*a*〉 slip systems. We employ these data in order to identify slip strengths at room temperature and these, together with the other properties, are shown in table 1 and the corresponding comparisons between single-crystal measurements and crystal model calculations for prismatic and *c*+*a* pyramidal slip are shown in figure 2. We note that the activation volume in the slip rule in equation (2.1) is defined as $V=\gamma_{0}b^{2}/\sqrt{\rho_{o}}$ which gives a value of 18*b*^3^, which is within the range given by Conrad [28] as 8*b*^3^–80*b*^3^ for alpha titanium. Equal strengths are assumed here for basal and prismatic slip systems for simplicity, since the difference between them has been observed to be negligibly small in experimental observations [13]. The slip strength of pyramidal systems is often observed to be about three times that for basal or prismatic systems, recently demonstrated experimentally in Gong & Wilkinson [25]. It is also reasonable to assume that there is no hardening occurring for the single-crystal titanium alloy response, and, as a result, the hardening coefficient *γ*^′^ is taken to be zero.

**Table 1.** Slip rule properties at 20°C and parameters (see text for temperature dependencies).

parameters	quantities
*ρ*_m_	5.0 μm^−2^
*ρ*_*o*_	0.01 μm^−2^
*v*	1.0×10^11^ Hz
*b*	3.20×10^−4^ μm
*k*	1.381×10^−23^ J K^−1^
*γ*_0_	6×10^−4^
*γ*^′^	0 for single crystal
	0.05 for polycrystal
*τ*^basal,prism^_c0_	280 MPa
*τ*^〈*c*+*a*〉^_c0_	840 MPa
Δ*H*	9.913×10^−20^ J atom^−1^

In order to establish the appropriate activation energy Δ*H* that enables the correct and consistent rate sensitivity in single-crystal and polycrystalline tests to be reproduced, the polycrystal data of Hasija *et al.* [13] are used in combination with a polycrystal model with approximately random texture and subjected to the equivalent strain-controlled mechanical loading. It is noted that in low temperature creep, i.e. less than 0.25 *T*_m_ (melting temperature), primary transient creep dominates over steady-state deformation. Steady-state creep of titanium alloy is often not observed in titanium alloys at room temperature [20,29,30]. The activation energy in transient creep is usually smaller than that measured in steady-state creep [20,31,32]. These observations are closely related to the strain rate sensitivity at room temperature [33,34] behaviour.

**Figure 3.**
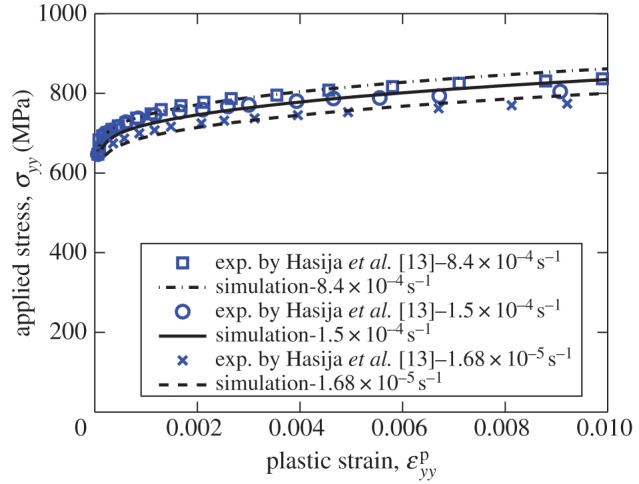
Strain rate sensitivity calibration based on polycrystalline experiments.

In addition, experimental studies in Ti-6Al alloy showed the transient creep was highly correlated to the short-range order (SRO) of titanium and aluminium atoms [30], strongly influencing strengthening and creep transients [35]. The polycrystal model rate sensitivity with an activation energy Δ*H*=9.91310^−20^ J/atom is shown for the three strain rates indicated, providing a good representation of the polycrystal average stress–strain response, in figure 3. The small amount of strengthening observed is captured simply by means of the contribution from Taylor hardening, such that the hardening factor, *γ*^′^, becomes non-zero (*γ*^′^=0.05). The slip rule properties at 20°C and parameters, the latter including initial densities of SSDs and GNDs, representative shear strain *γ*_0_, and the activation energy Δ*H*, are summarized in table 1.

**Figure 4.**
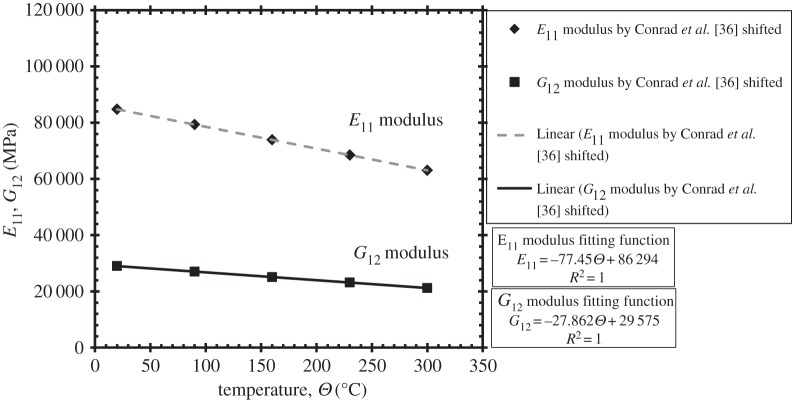
Temperature-dependent Young's modulus and shear modulus.

The temperature dependences are considered next. Figure 4 shows the temperature sensitivity in moduli determined by Conrad *et al.* [36], but the absolute values of the moduli have been shifted in order to ensure that the room-temperature moduli equate to those determined by Hasija *et al.* [13] to provide equivalence with their data for Ti-6Al. Full details of the elastic anisotropy for transversely isotropic HCP crystals and the corresponding elastic stiffness matrix are given in Dunne *et al.* [12], and the temperature dependencies are shown in table 2.

**Table 2.** Parameters for polycrystalline Ti model under uniaxial loading and room temperature.

	moduli (MPa) and Poisson's ratio					
temperature (°C)	*E*_11_	*E*_33_	*G*_12_	*G*_13_	*v*_12_	*v*_13_
20	84 745	119 789	29 022	40 000	0.46	0.22
90	79 323	112 125	27 065	37 303	0.47	0.22
160	73 902	104 462	25 113	34 612	0.47	0.23
230	68 480	96 798	23 164	31 926	0.48	0.23

**Figure 5.**
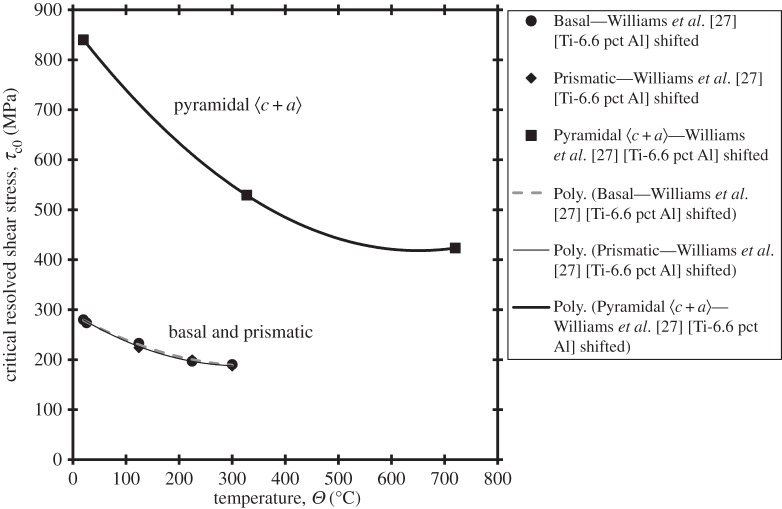
Temperature dependence of critical resolved shear stress for basal, prismatic and pyramidal 〈*c*+*a*〉 slip systems. That data referred to as ‘shifted’ is simply the original measured data of Williams *et al.* [27] translated vertically to ensure that the 20°C slip strengths are the same as those of Hasija *et al.* [13].

In the slip rule, the key properties with temperature dependence are the critical resolved shear stresses. The conjugate representative shear strain, *γ*_0_, is temperature independent, and the hardening coefficient *γ*^′^ is negligible or very small even at 20°C, so is considered independent of temperature. The activation energy Δ*H* is thought to be temperature independent in low temperature creep [19,20], and hence this is imposed here. The temperature *sensitivity* of the critical resolved shear stresses is obtained based on the reported experimental data by Williams *et al.* [27] and Hasija *et al.* [13]. However, there is not complete consistency between the sets of data, and because the slip rule rate sensitivity is calibrated against the polycrystal data of Hasija, we use the temperature *sensitivity* of slip strengths obtained by Williams *et al.*, while maintaining the 20°C absolute values of slip strengths from Hasija *et al.* In this way, the slip strengths at 20°C shown in figure 5 reproduce the single-crystal Ti-6Al test data of Hasija *et al.* [13]. The basal and prismatic slip strengths are assumed to take the same temperature sensitivity since their values are near-identical over the temperature range of interest, also shown in figure 5, and their temperature dependencies are given by

2.7 $$\tau_{c0}^{b,p}=0.0009\Theta^{2}-0.5942\Theta +290.55,$$

where *Θ* is the temperature between 20°C and 300°C. Similarly, the temperature dependence of the critical resolved shear stress for the pyramidal 〈*c*+*a*〉 systems, also shown in figure 5, is given by

2.8 $$\tau_{c0}^{\left\langle c+a\right\rangle }=0.0011\Theta^{2}-1.3801\Theta +867.18.$$

In the next section, the fully calibrated crystal model is employed to examine single-crystal Ti-6Al response to both strain and stress-controlled loading in order to establish bounds for the likely polycrystal stress relaxation and creep response.

## 3. Monotonic stress relaxation and creep in single-crystal behaviour

**Figure 6.**
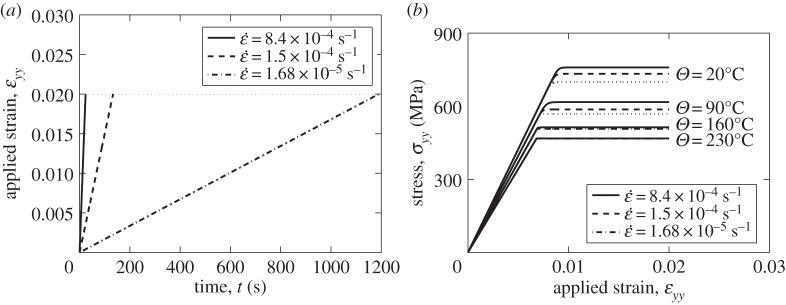
Linear strain loading with different rates: single crystal with prismatic slip.

Single-crystal prismatic slip under monotonic strain-controlled loading is considered first, for a range of strain rates and temperatures, particularly in order establish the expected bounds on temperature sensitivity. Figure 6*a* shows the strain applied up to value of 0.02, to the single crystal at the three strain rates $\dot{\varepsilon }=8.4\times 10^{-4},1.5\times 10^{-4},$ and 1.65 ×10^−5^ s^−1^ shown. Figure 6*b* shows the corresponding calculated loading-direction stress versus strain response for the range of strain rates and temperatures shown. With increasing temperature, so the modulus reduces, but the fully calibrated rate sensitivity shows more interesting results. That is, the room-temperature stress response shows the expected single-crystal reproduction of the sensitivity to strain rate, even at 20°C, showing about an 80 MPa change over the strain rate range. However, as the temperature is increased up to 230°C, the manifestation of strain rate sensitivity is seen to diminish, but in fact this results from the stronger thermal activation at higher temperature leading to very much more rapid stress relaxation, hence diminishing the stresses achieved even at the higher rates. This behaviour is anticipated in rate-sensitive materials (often at homologous temperatures in excess of about 0.4), but perhaps less so in relatively low temperature Ti alloy response.

**Figure 7.**
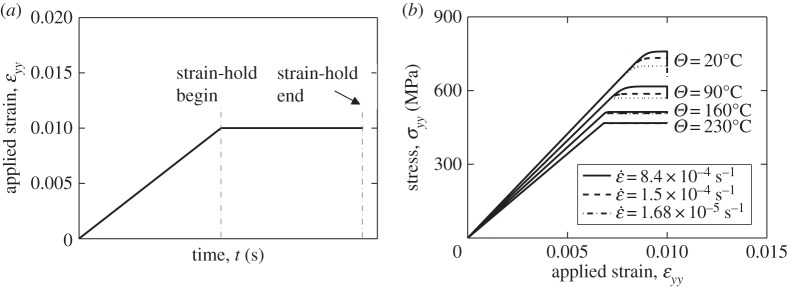
Dwell strain loading with different rates: single crystal with prismatic slip.

Introducing a strain-hold period in to the loading allows the resulting temperature dependence of the stress relaxation to be examined. The strain-controlled loading is shown in figure 7*a*, in which a maximum strain of 0.01 is imposed, followed by the strain-hold. The stress relaxation which results at 20°C shown in figure 7*b* can be seen to be significant and is retained for a temperature of 90°C but is seen to diminish to near-zero at temperatures in excess of about 160°C. This is a further manifestation of the thermal activation in that as the temperature increases, so does the diffusion-induced recovery reflected in more rapid stress relaxation, to the extent that there is no observable difference in stress response, at least for the strain rate range chosen, for temperatures above 160°C. There is clearly a close coupling between strain rate and temperature, and it is argued that this is likely to be profound in its consequences for cold dwell fatigue, and its temperature sensitivity, and this is addressed in detail in a later section. Firstly, however, as a next step, we address polycrystal response to strain-and stress-controlled loading with load holds.

## 4. Stress relaxation and creep in Ti-6Al polycrystal response

**Figure 8.**
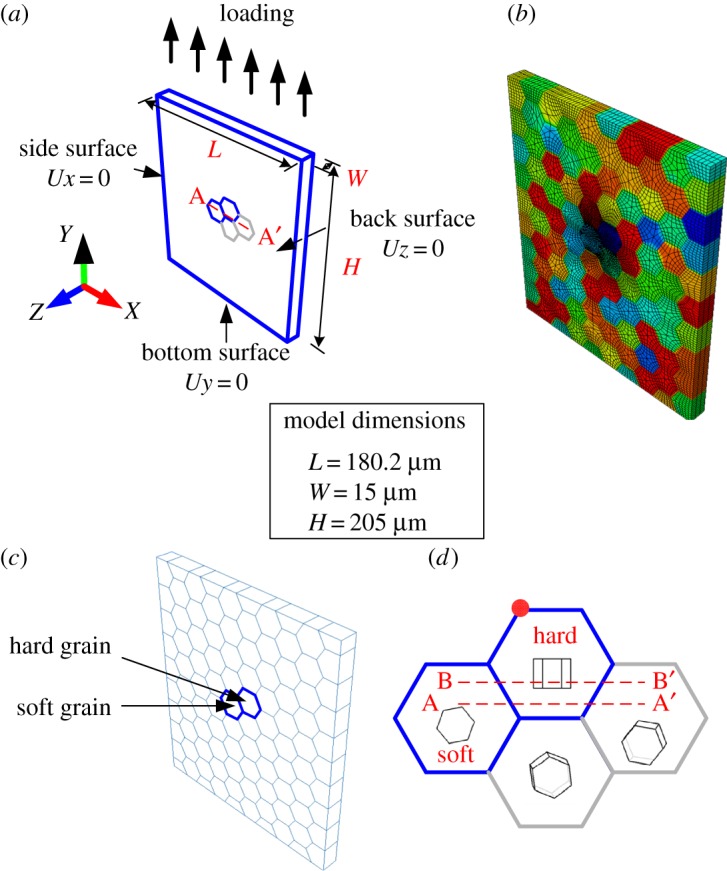
Polycrystalline finite-element model: (*a*) loading and boundary conditions; (*b*) polycrystalline model with 1625 element for the hard grain, 1637 elements for the soft grain and about 120 elements for all other grains; (*c*) location of rogue grain combination of hard and soft grains and (*d*) crystal orientations in and around the rogue grain combination, and the locations of the A-A' and B-B' paths as well as hotspot identification for local stress and strain analysis.

Polycrystal behaviour is assessed using the three-dimensional representation shown in figure 8. Grains with hexagonal morphology are considered, with pseudo-directionally solidified structure such that grains are prismatic both in shape and crystallographic orientation.

As shown in figure 8, the polycrystalline finite-element model developed has dimensions of 15×180.2×205 μm with a total of 300 grains each of about 25 μm in size. A rogue grain combination is considered in the central region of the polycrystal model because of its importance for cold dwell facet fatigue nucleation [11,37,38]. The model considered contains about 44 000 ABAQUS user-defined 20-noded solid elements, but for the central rogue combination shown in figure 8*c*, the hard and soft grains identified each contain about 1625 elements, while other grains each contain about 120 elements. The crystallographic orientations in the rogue combination are indicated schematically in figure 8*d*, in which the *c*-axis of the hard grain is orientated along the loading direction, and that of the soft grain is normal to the loading direction, and out of plane so that prismatic slip is likely to be activated. The two additional grains adjacent to the hard grain are also orientated to be easy for slip, but the remaining grains shown in figure 8*b*, are assigned random orientations. The polycrystal is subjected to uniaxial loading with boundary conditions indicated in figure 8. The crystal properties are those discussed above and given in tables 1 and 2.

### (a) Stress relaxation under strain control

**Figure 9.**
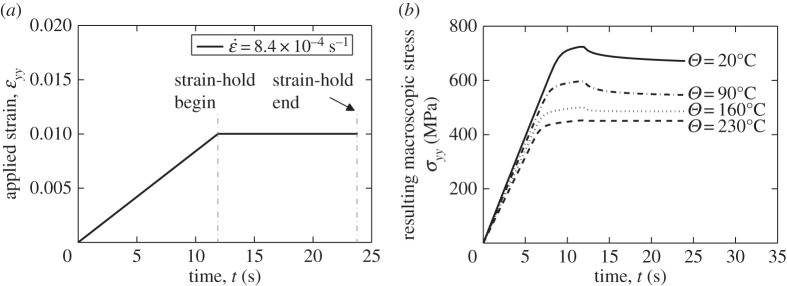
Macroscopic stress relaxation in polycrystalline model: (*a*) dwell strain loading; (*b*) *yy* stress response at the temperatures shown.

In this study, we choose to focus on the loading strain rate of $\dot{\varepsilon }=8.4\times 10^{-4}\;{\;s}^{-1}$ since this gives rise to a range of differing stress relaxations over the temperature range of interest 20–230°C. The macro-level strain-controlled loading applied is shown in figure 9*a* which includes a strain-hold period leading to the corresponding macro-level stress response shown in figure 9*b*. It is noted that the overall polycrystal response is very similar to that observed for the single-crystal behaviour such that pronounced stress relaxation is seen during the strain-holds at lower temperature, but as the temperature increases to about 230°C or above, the relaxation takes place much more rapidly by virtue of thermal activation such that for the strain rate loading considered, at the higher temperatures, the stress relaxation occurs over a small timescale compared with the loading time.

**Figure 10.**
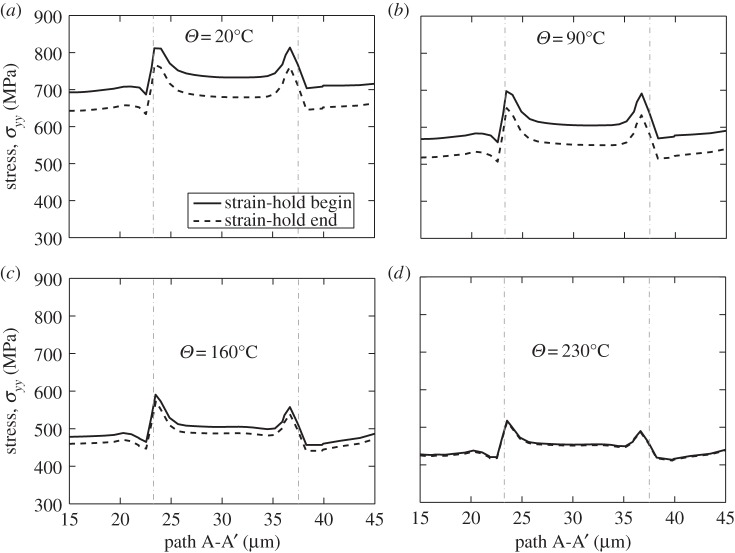
Stress relaxation in path A-A' at elevated temperatures: (*a*) *Θ*=20°C, (*b*) *Θ*=90°C, (*c*) *Θ*=160°C and (*d*) *Θ*=230°C.

It is helpful to study the local grain-level behaviour at the rogue grain combination along path A-A' through the rogue grain as indicated in figure 8*a*,*d*. The local *yy*-stress responses at the strain-hold start (*t*=12 s) and at the strain-hold end (*t*=24 s) are shown in figure 10 from 20°C to 230°C for the Ti-6Al polycrystal considered. The boundaries at the soft–hard and hard–soft grains are indicated by the dashed lines shown in figure 10. The peak stresses which occur at the boundaries of hard and soft grains are due to the neighbour effects indicated in figure 8*d*. A steady decrease of *yy*-stress along path A-A' (indicated in figure 8*d*) from a peak at the grain boundary of about 800 MPa at 20°C in figure 10*a* to just over 500 MPa at 230°C in figure 10*d* may be seen. This results largely from the slip strength dependence on temperature shown in figure 5; it is noted in passing that the magnitude of the decrease in stress for this near-alpha (Ti-6Al) alloy is larger than would be expected to occur in more conventional, industrial Ti alloys. The figures also show the stresses both at the beginning and end of the strain-hold period, as indicated in figure 9*a*, and what is of particular interest is that the magnitude of the stress relaxation which appears to occur progressively diminishes with increasing temperature such that at 230°C, the local stresses both before and after the strain-hold period are near-identical. This again results from the thermally activated creep taking place in the grains well-orientated for slip, and relates directly to the activation energy term, Δ*H*, in the slip rule in equation (2.1), reflecting the strain rate, or creep sensitivity to temperature.

### (b) Creep and load shedding

It is recognized that cold dwell fatigue is known to be more damaging under loading conditions of stress-hold than for those of strain-hold [11], hence it is important and useful to examine the very same polycrystal containing the rogue grain combination of crystallographic orientations but with stress-hold loading conditions reflecting the experimentally observed worst case.

**Figure 11.**
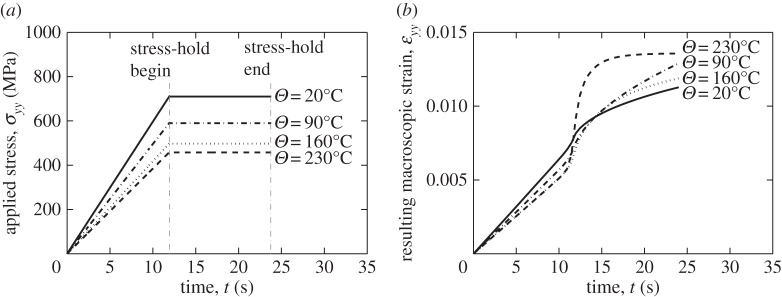
Stress-hold in polycrystalline model material: (*a*) macroscale *YY* stress, and (*b*) the resulting macroscale strain response.

The stress-controlled loading conditions imposed are shown in figure 11*a*. The total loading time is again 24 s, with equal periods for load-up and stress-hold. The holding stress magnitudes applied are 711, 590, 497 and 458 MPa for 20, 90, 160 and 230°C, respectively, chosen to ensure that plasticity develops at the soft–hard grain boundary in all cases. In particular, because of the relatively high yield strain in the titanium alloy considered, we have chosen to apply a loading stress at each temperature of interest in order to ensure a macro-level applied strain of about 1% consistently to ensure that local yielding occurs at the hard–soft grain boundary. Hence, we are able to make comparisons consistently across the temperature range considered. The resulting macroscale *yy* strain responses are shown in figure 11*b*, which indicate an initial elastic linear response, followed by plastic straining and finally, the accumulation of strain through creep deformation. An interesting observation is that for the case of 230°C, the strain develops rapidly during the initial part of the stress-hold and finally saturates at a value of about 0.0136. This strain saturation phenomenon is not observed for other temperatures for these conditions of loading rate. Also, in passing, it is noted that the rate of strain accumulation at the termination of the stress-hold period is highest, for the discrete temperatures considered, at the temperature of 90°C; we return to this interesting result later in the context of dwell fatigue sensitivity to temperature, and load shedding, which are addressed next.

#### (i) Load shedding

**Figure 12.**
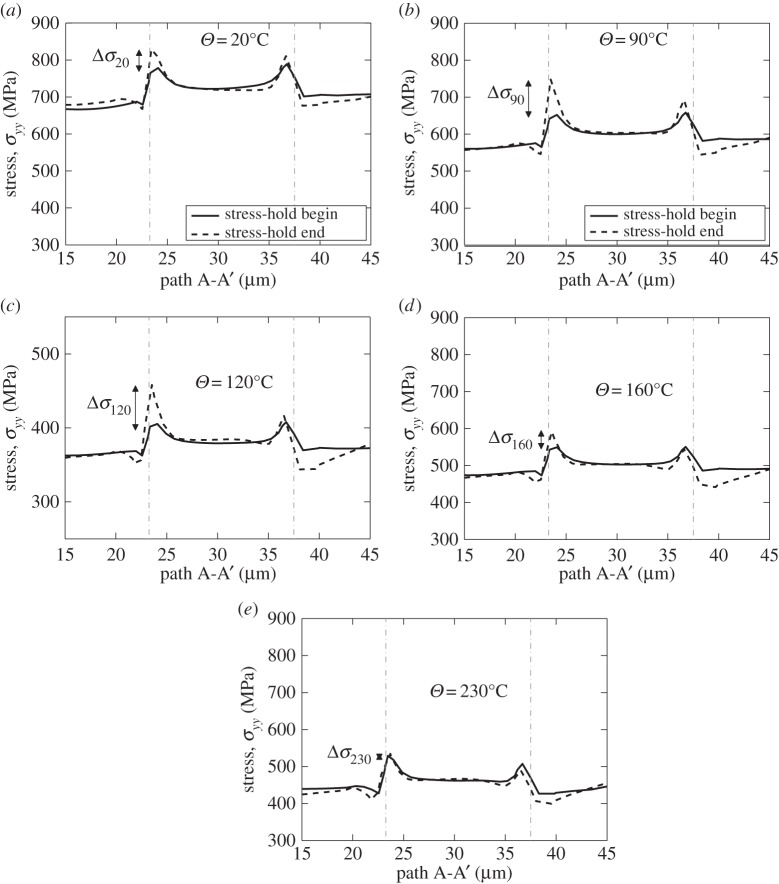
Stress *YY* along path A-A' for increasing temperatures: (*a*) *Θ*=20°C, (*b*) *Θ*=90°C, (*c*) *Θ*=120°C, (*d*) *Θ*=160°C and (*e*) *Θ*=230°C.

Load shedding was first identified as a potential key mechanism for the formation of cold dwell facets by Hasija *et al.* [13] and Venkataramani *et al.* [39,40], which occurs by local creep deformation in grains well-orientated for slip and adjacent to those badly orientated (e.g. in the rogue grain orientation configuration discussed above) such that the stress relaxation occurring in the creeping, soft, grains leads to the redistribution of the stress to the adjacent hard grain, which occurs during a cold dwell or stress-hold period. The effect is shown clearly in figure 12 but now over the range of temperature of interest. Here, the very same stress-controlled loading shown in figure 11*a* resulting in the macroscale strain responses in figure 11*b* leads to the local load shedding observed at the grain level in figure 12 which shows the stresses along path A-A' (figure 8*d*) during the load-up and macroscale stress-hold for the temperatures shown. In figure 12 again, the vertical broken lines indicate the locations of grain boundaries along path A-A'. The results show the role of load shedding which causes the stresses at the boundary between the soft and hard grains to increase significantly during the load dwell; figure 12 shows the *yy* stresses along path A-A' at the points for both stress dwell start and its end, shown in figure 11*a*. It is interesting to see that the magnitude of the stress redistribution due to load shedding is maximum at a temperature of 120°C, shown in figure 12*c*. This is higher than that observed at 20°C (though the stress redistribution at this temperature remains highly significant), but as the temperature increases further, so the magnitude of the stress redistributed during the hold period diminishes such that once a temperature of 230°C is considered, the stresses at the beginning and end of the load hold are largely the same. Two key points are therefore argued on the basis of the creep sensitivity to temperature in the Ti-6Al alloy considered. Firstly, the crucial role played by load shedding in cold dwell fatigue is seen to diminish and vanish in effect at a temperature of about 230°C; it is therefore of significant note that aero-engine manufacturer experience is such that the phenomenon of cold dwell fatigue and the dwell debit in disc components is known to diminish and vanish as temperatures approach about 200°C (D Rugg 2014, personal communication). Secondly, it is also industrial aero-engine experience (D Rugg 2014, personal communication) that the worst-case situation for dwell debit recorded in tests in commercial Ti alloys in discs occurs for a temperature at about 120°C; this is, of course, very close to the temperature for which the magnitude of the predicted stress redistribution is shown to be highest, and it is therefore important to establish the mechanistic basis for the temperature sensitivity of load shedding, and the temperature for which it is strongest, and this is addressed next.

#### (ii) Temperature sensitivity of load shedding

**Figure 13.**
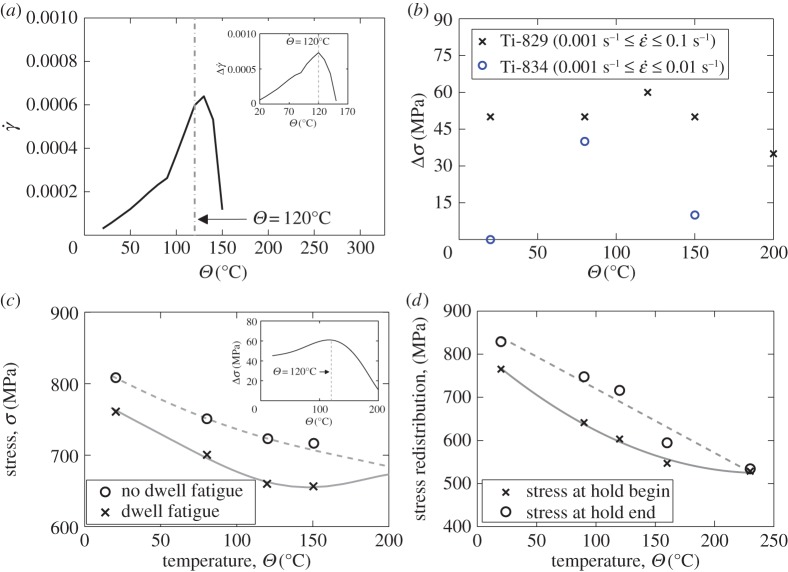
(*a*) Slip rate development with temperature in a creeping soft grain adjacent to a hard grain as shown schematically in figure 8; the insert figure shows the difference of slip rate from stress-hold begin to stress-hold end. (*b*) The maximum variation in 0.2% proof stress with temperature for the two alloys shown for the strain rate ranges indicated. (*c*) Experimental dwell and no-dwell fatigue endurance data (B Arthurs & A Walker 2014, personal communication) for Ti-829 alloy for the temperature range shown (inset shows stress difference with temperature). (*d*) Predicted stress redistribution (illustrated in figure 12) at hard–soft grain boundary during stress-hold.

Figure 12 indicates a temperature sensitivity of the magnitude of the load shedding; of the five temperatures analysed, the strongest load shedding was observed at 120°C. We note that the rate sensitivity in the material is captured through the activation energy term, Δ*H*, appearing in the slip rule in equation (2.2). Its sensitivity to temperature is captured because of the explicit representation of temperature both in the exponential and sinh terms resulting from the thermal formulation developed in [10,12,16,17] and appearing in the slip rule in equation (2.1). In addition, the slip strengths are also dependent on temperature as shown by equation (2.2), and also explicitly dependent upon the development of local dislocation density generation for differing slip systems, which in turn affect the slip rate in equation (2.2). In order to investigate the temperature sensitivity of load shedding, we therefore examine the slip rates, ${\dot{\gamma }}^{\alpha }$, developing local to the hard–soft rogue grain combination, since these control the rate and magnitude of the creep taking place in the soft grain, and, correspondingly, the stress relaxation and redistribution to the adjacent hard grain. Hence, returning to figure 8, under conditions of stress-hold loading, we examine the slip rates at the hard–soft grain boundary along path A-A' but just within the soft grain, over the range of temperatures of interest and the results are shown in figure 13*a*. It is found that a peak slip rate exists (indicating highest propensity for creep and therefore load shedding) at a temperature of about 120°C. Also, the insert in figure 13*a* shows the change in slip rate $\Delta \dot{\gamma }$ between the start and finish of the dwell period of loading. Thus, the peak in load shedding with temperature in figure 12 is attributed to the slip rate dependence on temperature, and it is intriguing to note that the peak slip rate which gives rise to the strongest load shedding occurs at 120°C and coincides with the anecdotally observed in-service worst-case dwell debit temperature (D Rugg 2014, personal communication). In addition, independent rate sensitivity data for a Rolls-Royce near-alpha alloy Ti-829 (B Arthurs & A Walker 2014, personal communication) given in figure 13*b* shows that the highest measured rate sensitivity of 0.2% proof stress in these alloys over the temperature range 20–300°C also occurs at 120°C, therefore supporting the results of the calculations. Experimental data for Ti-834 also shown do not contradict this result. Additional experimental data exist (B Arthurs & A Walker 2014, personal communication) for alloy Ti-829 comparing the fatigue endurance stress (10^4^ cycles) for non-dwell and dwell (with a 2 min stress-hold per cycle) fatigue over temperatures from 20°C to 200°C. These results are shown in figure 13*c* with the stress (for life of 10^4^ cycles) decrease resulting from the inclusion of the dwell shown in the inset. It is clearly seen that the largest detriment to endurance under dwell conditions occurs at 120°C, and that the dwell effect diminishes progressively at temperatures above this. Both of these independent experimental results show direct and unambiguous agreement with the model predictions.

Analysis of the slip rule in equation (2.2), for the case of fixed slip strength with temperature, *τ*^*α*^_c0_(*Θ*), indicates that slip rate is anticipated to increase monotonically with temperature and that therefore the behaviour shown in figure 13*a* deviates from this expectation. But in fact, during the load hold, the soft grain undergoes creep by slip, leading to the development of dislocation activity which increases the slip strength as shown in equation (2.2), and the behaviour shown in figure 13*a* results interestingly because of the close coupling between slip rate and slip strengthening, which is itself dependent on temperature.

The stress redistribution resulting from load shedding at the hard–soft grain boundary is extracted for all temperatures with reference to figure 12*a*–*d*, and the grain boundary stresses at the beginning and end of the hold period are shown in figure 13*d* for increasing temperature. The differences, Δ*σ*, shown in figure 12*a*–*d* correspond to the load shedding, or stress redistribution resulting from the hold period, with the maximum occurring at 120°C. At 230°C, the load shedding is seen to diminish to zero. In addition, the nature of the stress difference shown in figure 13*d* over temperature shows a striking relationship with the experimentally observed stress dwell debit given in figure 13*c*.

#### (iii) The role of the stress relaxation in cold dwell

An important issue for cold dwell fatigue debit in service applications is the representative time scale over which cold creep takes place. Put another way, the time constant associated with stress relaxation is crucial because of the relevance of the period of time for which the most damaging combination of component-level stress and temperature occurs in service. Hence in passing, because the crystal model has been calibrated against rate-sensitive data, we examine the stress relaxation response to dwell and its associated time scales.

**Figure 14.**
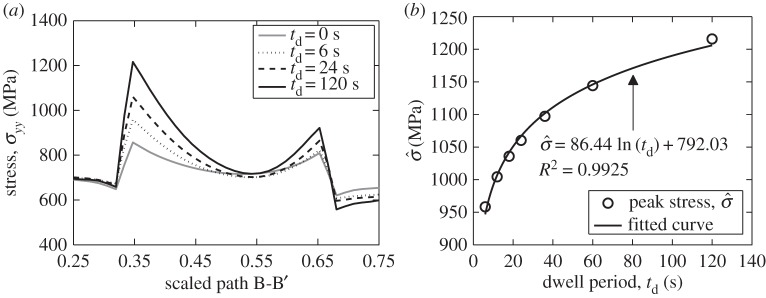
Peak stress at soft–hard grain boundaries with increasing dwell period: (*a*) local stress along path B-B' and (*b*) peak stress in the path B-B' with varying dwell period.

In order to show the critical peak stress along the soft–hard grain boundaries, we shift path A-A' up to the centre height of the grains and refer to the new path as B-B', as shown in figure 8*d*. Recalling the dwell stress loading curve in figure 11*a*, the dwell period *t*_*d*_ now considered is measured from the stress-hold point (*t*=12 s). The stresses along path B-B' are shown in figure 14*a* and are extracted at various values of dwell period *t*_*d*_. For illustrative purposes, the peak boundary stress is included in figure 14*b*; this demonstrates the increase in stress at the soft–hard grain boundary. Note also that this particular study was carried out at room temperature (20°C).

This result agrees well with Hasija *et al*. [13], who first noted that local peak boundary stress is a function of time and also echoes the study by Bache *et al*. [41], in which the effect of increasing dwell period led to a decrease in the number of cycles to failure in a tensile dwell fatigue sample. In passing, it is worth noting that an in-service dwell period may be approximately 120 s (B Arthurs & A Walker 2014, personal communication) and a suitable value for critical cleavage stress is approximately 1200 MPa [11,42]. This would suggest that under an applied stress of 711 MPa, temperatures in the range of 20–80°C coupled with a dwell period of 120 s or above may lead to facet nucleation.

#### (iv) The role of strain accumulation in cold dwell

**Figure 15.**
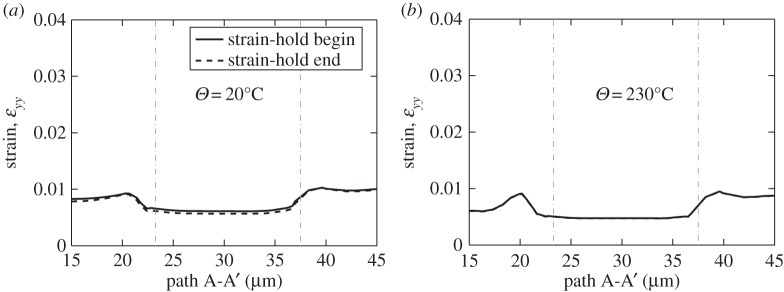
Local strain along path A-A' under strain-controlled loading and a strain-dwell at (*a*) *Θ*=20°C and (*b*) *Θ*=230°C.

When focusing on the grain-level deformation in the cases of both strain and stress-controlled dwell cycling, it has been argued that a stress-hold is more damaging than that for strain, and perhaps more importantly, this is also the evidence from laboratory and in-service behaviour [11]. This phenomenon is investigated here by consideration of the local grain-level, rate-dependent deformation taking place in both loading types. As before, the representative model polycrystal shown in figure 8 is employed, subjected to the strain and stress-hold loading discussed above and, as shown in figure 15*a* for 20°C, the resulting local strain along path A-A' does not show significant change or accumulation during the dwell period for strain-controlled loading. The result for the same loading but at a temperature of 230°C is shown in figure 15*b*, indicating that even for potentially very much more thermally activated slip, the strain accumulation under this loading remains low such that consequently, the stress relaxation and hence load shedding are also minimal. Ultimately, the result indicates that load shedding and a corresponding dwell debit are inhibited at the rogue grain combination for strain-controlled loading.

## 5. Cyclic polycrystal behaviour with relaxation and ratcheting

**Figure 16.**
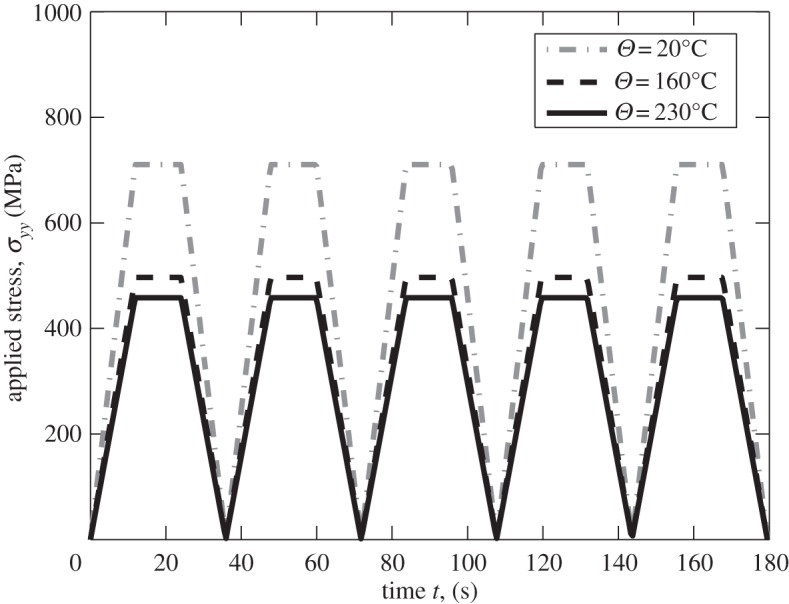
Cyclic loading with stress dwell for the stresses and temperatures shown.

Dwell fatigue is primarily a cyclic process and while great insight at the microstructural level in terms of microcreep, stress relaxation and load shedding may be obtained from analyses of a single cycle stress dwell, these effects are also important in determining the cyclic response. Notably, loading for which a non-zero mean stress is applied often leads to the progressive accumulation of plastic strain, or ratcheting. This may occur both at the microstructural level (e.g. [43]), and at the macro-level, and this section addresses polycrystal creep, stress relaxation, ratcheting, and elastic and plastic shakedown, under cold dwell cyclic loading. Stress-controlled cycling incorporating the stress dwell is considered over the temperature range as shown in figure 16 and applied to the polycrystal model shown in figure 8.

**Figure 17.**
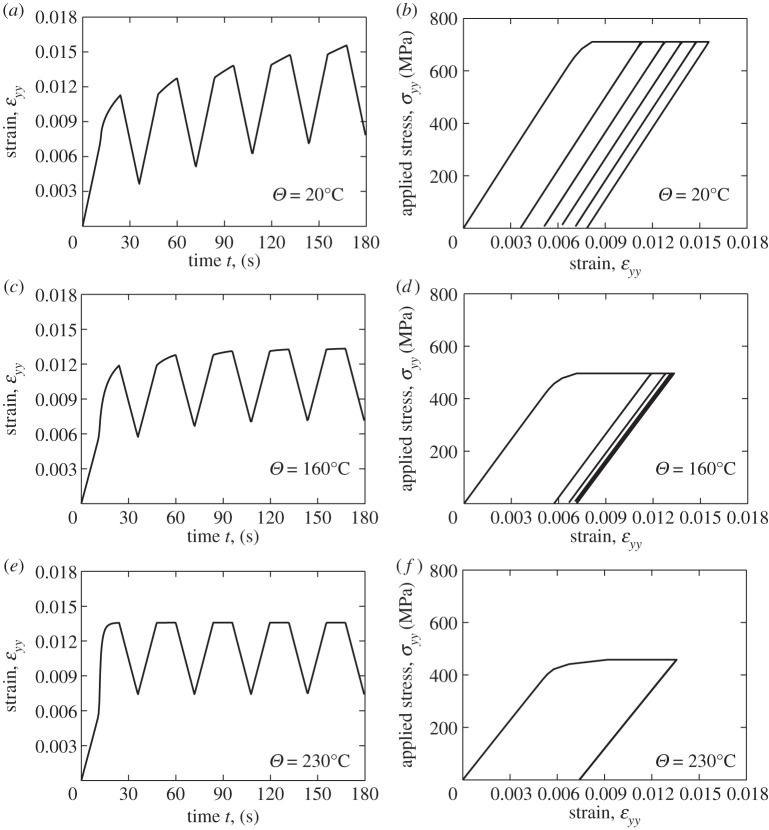
Macroscopic strain response and stress–strain behaviour for increasing temperature.

The macroscale strain response and the corresponding stress–strain behaviour which result for the polycrystal are given in figure 17. At a temperature of 20°C, the macroscale strain is seen to increase progressively with cycles but at reducing rate per cycle, such that the resulting macroscale stress—strain hysteresis response suggests the onset of plastic shakedown, leading to a subsequent constant strain increment per cycle. The corresponding result for 160°C (at a lower applied stress) also results in ratcheting but the accumulated strain within the first cycle is larger because of the enhanced creep rate, and subsequent plastic shakedown occurs more rapidly than for 20°C. A more extreme response is observed for 230°C (at lower applied stress again) in which the creep rate is so high that within the first cycle, the strain accumulation saturates, leading to no further ratcheting nor cyclic plasticity, and elastic shakedown takes place. Over really quite a narrow range of temperature, the remarkable low-temperature rate sensitivity of Ti-6Al alloy gives rise to a broad range of cyclic responses from cyclic plasticity and ratcheting through to elastic shakedown at the macroscale.

**Figure 18.**
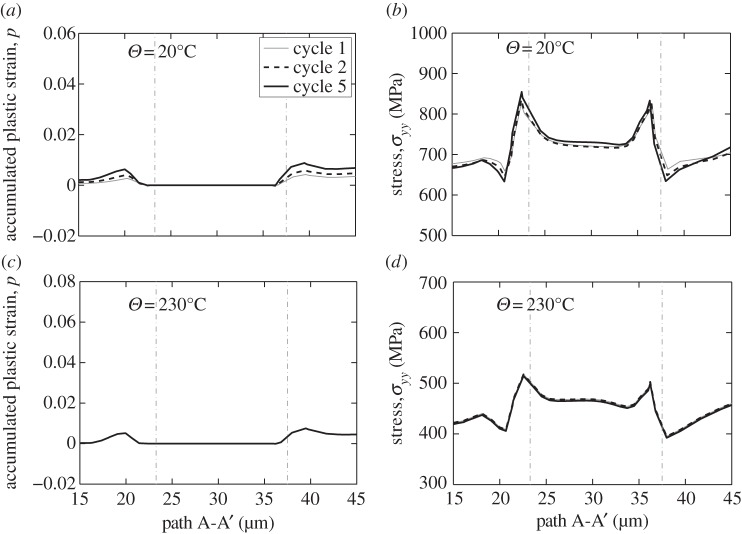
Microscale plastic strain and stress along path A-A' at the end of stress dwell loading at the cycles indicated for the temperatures shown.

**Figure 19.**
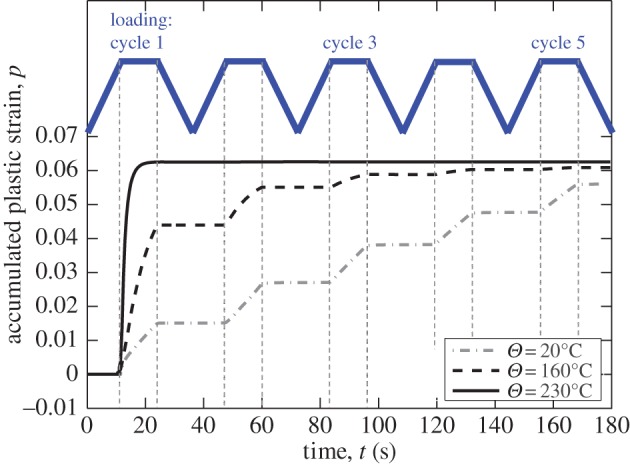
Accumulated plastic strain at the hard–soft grain interface shown in figure 8*d*.

The microscale or grain-level response is considered at the location of the rogue grain combination in figure 8 for the same loading conditions at the end of the stress dwell but for the two temperatures of 20°C and 230°C; that is, for the lower temperature for which load shedding was demonstrated earlier to be significant, and for a higher temperature for which creep and stress relaxation occurs so rapidly that no load shedding during the stress-hold is observed at all. The key results along path A-A' are shown in figure 18 for loading cycles 1, 2 and 5 and have significant implications for cold dwell fatigue. Figure 18*a*,*b* shows the room-temperature results in which cold creep in the soft grains gives rise to the progressive cyclic accumulation of plastic strain over just five cycles which results in the cyclic redistribution of stress from the soft grain to the adjacent hard grain; it is interesting to note that this is a *cyclic* phenomenon as opposed to that due to dwell, but one that similarly results from the low-temperature rate sensitivity of this material. This is demonstrated in figure 19 in which the accumulated strain at a particular hard–soft grain interface, identified in figure 8*d*, is shown with loading cycles where it is seen to be the case that the strain accumulation occurs only during the stress-hold. That is, the local ratcheting occurs primarily through rate-dependent slip, or creep processes, and not from rate-independent reversed plasticity.

Figure 18*c*,*d* shows the corresponding results for the higher temperature of 230°C in which it is seen firstly that the plastic strain accumulation in the soft grains occurs within the first loading cycle and that its magnitude is significantly higher than that for the lower temperature and, that very limited further accumulation with subsequent cycles occurs. Secondly, a consequence of this is that there is no cyclic load shedding observed in figure 18*d* at this temperature such that a cyclic, progressive increase in the stress carried by the hard grain does not occur. This result further supports the hypothesis presented earlier that temperatures in excess of 230°C are likely to inhibit the conditions necessary in order to generate hard-grain stresses sufficiently large in order to nucleate facets.

Hence, under dwell fatigue loading, there exists the progressive cyclic shedding of stress from the soft grain to the hard grain, which takes place in addition to the load shedding resulting solely from the stress dwell. The existence of the additional cyclic load shedding, over and above that due to dwell load shedding, provides a rational basis for the cycle dependence of the nucleation of dwell facets, and provides a potential explanation for the incubation period necessary in order for fatigue facets to nucleate.

## 6. Conclusion

The temperature sensitivity of load shedding and cold dwell has been investigated in model alloy Ti-6Al, and it is shown that load shedding is crucially dependent on temperature from 20°C up to about 230°C. In temperatures in excess of the latter, thermally activated creep leads to very rapid stress redistribution and the diminution, and in effect elimination, of local load shedding from soft to adjacent hard grains. It is therefore argued that the well-known cold dwell fatigue debit in Ti alloys is likely to diminish to zero at temperatures in excess of about 230°C. It is also shown that an intermediary temperature of about 120°C exists for which stress relaxation is the strongest. This results from a coupling between slip rate and temperature-dependent dislocation strengthening. Hence the worst-case dwell fatigue debit is expected to be observed at about 120°C. Anecdotal industrial evidence supports this result, as well as the experimental observations of highest rate sensitivities at this temperature in two Ti alloys Ti-829 and Ti-834. In addition, experimental studies of dwell debit in Ti-829 have shown unambiguously the debit to be worst at 120°C, and that it diminishes to a small level with temperatures in excess of 200°C, thus providing strong support for the mechanistic basis presented. The dwell period, crucial for determining the time constant associated with load shedding, is predicted to be of order minutes rather than seconds; this is likely to be of significance for aero-engine flight loading cycles.

Analysis of slip at hard–soft grain interfaces shows that under strain-controlled loading holds, local rate-dependent slip accumulation does not occur, so inhibiting stress relaxation and the shedding of load to the adjacent hard grain. Conversely, for conditions of stress-controlled dwell loading, it is the soft grain rate-dependent slip accumulation during the dwell period which leads directly to stress relaxation in the soft grain and the corresponding load shedding. It is therefore argued that a stress dwell cycle is more damaging in dwell debit than that for strain-controlled dwell.

Cyclic loading containing stress dwell is shown to lead to the interesting process of cyclic load shedding at the hard–soft grain interface; that is, the progressive, cycle-by-cycle redistribution of stress from the soft grain to the hard grain. This occurs because of local, soft grain ratcheting, or progressive accumulation of slip, which occurs only during the stress-hold part of the cycle showing it to result solely from the material's rate sensitivity as opposed to the reversed plasticity. Hence, a rationale for a cyclic incubation period for facet nucleation has been presented, and it is argued that nucleation depends upon both the stress dwell load shedding and the *cyclic load shedding*.

## Data accessibility

This work relies on existing published and referenced experimental data, except that provided independently by Rolls-Royce plc, explicitly recognized in the acknowledgement.
